# S-equol producing bacteria: isolation and identification from Albino Wistar rat gut microbiota

**DOI:** 10.1007/s00253-026-13759-4

**Published:** 2026-03-02

**Authors:** Megha Gangwar, Sanaa Ismael Abduljabbar, Jalaluddin Khan, Mohammad Sarwar Alam, Kahksha Ahmed, Sameena Naaz, Bibhu Prasad Panda

**Affiliations:** 1 Department of Food Technology, School of Interdisciplinary Sciences & Technology, Jamia Hamdard, New Delhi, 110062 India; 2 Microbial and Pharmaceutical Biotechnology Laboratory (MPBL), Department of Pharmacognosy & Phytochemistry, School of Pharmaceutical Education & Research (SPER), Jamia Hamdard, New Delhi, 110062 India; 3 Department of Chemistry, School of Chemical and Life Sciences, Jamia Hamdard, New Delhi, 110062 India; 4 Department of Computer Science and Engineering, St. Andrews Institute of Technology & Management (SAITM), Gurugram, 122506 India; 5 Department of Computer Science, School of Arts, Humanities and Social Sciences, University of Roehampton, London, SW15 5PH UK

**Keywords:** Soy isoflavones, Daidzein, Equol, Gut bacteria

## Abstract

**Abstract:**

The metabolism of soy isoflavones by gut microbiota is critical for the bioactivation and bioavailability of these compounds, particularly daidzein, which is further metabolized by gut bacteria to produce S-equol. S-equol, an exclusive gut bacterial metabolite, is associated with health benefits such as reduced blood pressure, cardiovascular disease prevention, and protection against hormone-related cancers due to its estrogen-mimicking structure and antioxidant properties. However, the limited availability of S-equol-producing bacteria has hindered its production and utilization. This study investigates the isolation and characterization of S-equol-producing microbes from albino Wistar rats and explores the impact of dietary interventions on S-equol production. Preliminary tests showed that both dietary groups excreted more S-equol in feces than urine, with rats on fermented soy feed showing higher S-equol levels due to the presence of daidzein, a precursor. In this study, we isolated four anaerobic S-equol-producing bacteria — MG1 (PX459562), MG2 (PX459563), MG3 (PX459564), and MG4 (PX459565) from the intestine and feces of albino Wistar rats. High-Performance Thin-Layer Chromatography (HPTLC) and High-Performance Liquid Chromatography (HPLC) confirmed the presence of S-equol, with concentrations ranging from 5.90 to 7.56 µg/g of fermented soybean across different strains. Phylogenetic analysis revealed that the isolates belonged to the Enterobacteriaceae and Enterococcaceae families, identifying MG1 as *C. freundii* strain ATCC 8090, MG2 as *Escherichia fergusonii* strain NBRC 102419, and both MG3 and MG4 as *Enterococcus faecalis* strain NBRC 100480. Our findings underscore the significant role of gut microbiota in metabolizing daidzein into S-equol, highlighting the potential for utilizing these bacterial strains in functional food development and therapeutic applications. While the pathogenic nature of *E. fergusonii* (MG2) precludes its therapeutic use, strains MG1, MG3, and MG4, which match common commensal bacteria, show promise for commercial S-equol production and may serve as valuable resources for further investigation and utilization in promoting health and preventing associated diseases.

**Key points:**

• *Dietary intervention modulates gut microbiota in albino Wistar rats.*

• *Soybean fermentation enables efficient conversion of daidzin to bioactive S-equol.*

• *Novel S-equol–producing microbes were isolated and identified.*

**Graphical abstract:**

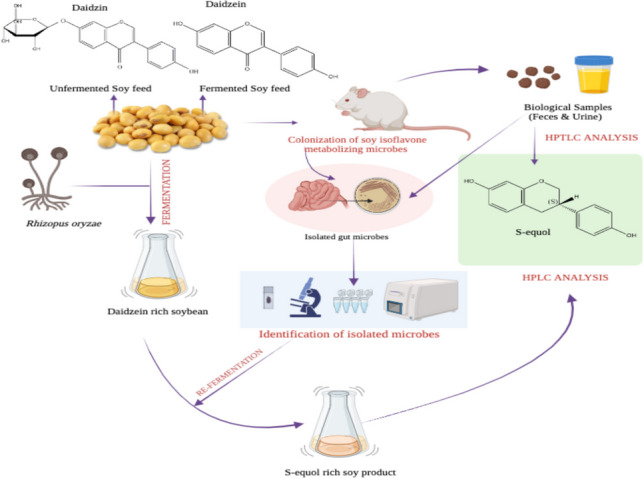

**Supplementary Information:**

The online version contains supplementary material available at 10.1007/s00253-026-13759-4.

## Introduction

For centuries, soy-based foods have been a dietary staple in Asian countries and continue to be commonly consumed today (Messina et al. 2022). Mean daily soy isoflavone intake among older adults in Japan ranges from approximately 30 to 50 mg/day, whereas intakes in the United States and Europe are typically much lower (<3 mg/day), with some regional estimates in Europe reaching approximately 7 mg/day (Messina et al. 2022). These differences in dietary exposure contribute to variation in the prevalence of equol-producing phenotypes across populations (Sekikawa et al. 2019). Soy foods are rich in the isoflavones daidzein and genistein, which have been associated with multiple health-promoting effects, including antioxidant, anti-inflammatory, anti-proliferative, and cardioprotective activities, as well as protective roles against osteoporosisand hormone-dependent cancers (Kao and Chen 2006; Kao et al. 2007a, b; Atkinson et al. 2005; Patisaul and Jefferson 2010; Aso et al. 2012; Kim 2021). However, accumulating evidence indicates that many of these beneficial effects may be mediated not directly by soy isoflavones themselves, but by S-equol, a gut microbial metabolite of daidzein with greater biological activity, including stronger antioxidant, anti-inflammatory, and estrogen receptor-β–selective effects (Sekikawa et al. 2022).

S-equol, an exclusive gut bacterial metabolite of the soy isoflavone daidzein, has gained considerable attention due to its potential health benefits. Equol exists as two enantiomeric forms, S-(–)-equol and R-(+)-equol, which differ in their spatial configuration and biological activity. Only the S-(–)-enantiomer is naturally produced in humans through intestinal bacterial metabolism of daidzein, whereas the R-(+)-form can be synthesized chemically. These two forms display distinct affinities toward estrogen receptors: S-(–)-equol preferentially binds to estrogen receptor β (ERβ) with higher potency, contributing to its estrogenic and antioxidant effects, while R-(+)-equol shows much weaker binding and limited biological activity (Setchell et al. 2002; Muthyala et al. 2004). Thus, when referring to “S-equol” in human studies, it specifically denotes the biologically active enantiomer derived from gut microbial conversion of daidzein.

S-equol is reported to lower the risk of cardiovascular diseases by reducing plasma levels of triglycerides and high-density lipoprotein (HDL) (Mayo et al. 2019). S-equol supplementation alleviates menopausal symptoms such as hot flushes and arterial stiffness (Jenks et al. 2012; Yoshikata et al. 2018). Administration of S-equol in postmenopausal women helps prevent osteoporosis by maintaining bone mineral density and suppressing bone resorption (Yoshikata et al. 2018). Additionally, S-equol shows potential in reducing the risk of prostate, colon, and breast cancers, although current evidence is still emerging, and further research is required to clarify its exact role (Fritz et al. 2013). Furthermore, S-equol is acknowledged for its diverse range of biological activities, encompassing antioxidative stress, anti-inflammatory, and antibacterial properties (Li 2019; Tanaka et al. 2019). It also holds potential for treating conditions such as atherosclerotic lesions and addressing major symptoms experienced by postmenopausal women (Chen et al. 2019; Yoshikata et al. 2021). S-equol has also shown promise in regulating obesity and type 2 diabetes (Charles et al. 2009; Usui et al. 2013) by controlling the glycemic index and alleviating chronic kidney disease (Jing and Wei-Jie 2016).

**Fig. 1:**
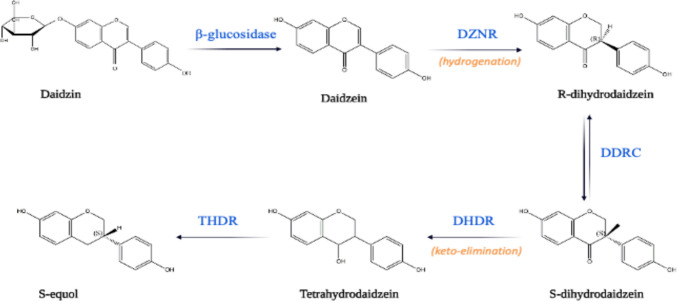
Metabolism of soy isoflavones by gut microbiota and biosynthesis of S-equol. S-equol, a biologically active metabolite of soy isoflavones, is produced in a multi-step enzymatic process catalyzed by gut microbiota. The biosynthesis of S-equol involves four key genes: daidzein reductase (DZNR), dihydrodaidzein racemase (DDRC), dihydrodaidzein reductase (DHDR), and tetrahydrodaidzein racemase (THDR). The metabolic pathway begins with daidzein, a isoflavone, which is metabolized into dihydrodaidzein through the combined actions of DZNR and DDRC. Subsequently, followed by the final conversion into S-equol facilitated by the enzymatic actions of DHDR and THDR

Biotransformation is a critical process that influences the biological effects of soy isoflavones found in our diet. These isoflavones are initially present as glycosides and must be converted into aglycones for optimal bioavailability through the activity of intestinal β-glycosidase from gut bacteria (Zubik and Meydani 2003). Once hydrolyzed, the aglycone form can be absorbed by the intestine or undergo further transformations by intestinal microflora. The conversion of daidzein to S-equol occurs through a three-step process (as shown in Fig. 1) (Hu et al. 2022) via dihydrodaidzein (DHD) and tetrahydrodaidzein (THD) (Kim et al. 2009). Research on S-equol-producing microbes has identified four crucial enzymes involved in its biosynthesis: daidzein reductase (DZNR), dihydrodaidzein racemase (DDRC), dihydrodaidzein reductase (DHDR), and tetrahydrodaidzein reductase (THDR) (Hu et al. 2022). S-equol is the final product of daidzin biotransformation, and once produced, it remains stable and does not undergo further metabolic changes.

Earlier hypotheses proposed that the complete biotransformation of daidzein to S-equol might require the coordinated activity of multiple intestinal bacterial species, with different taxa contributing individual enzymatic steps. However, recent review studies have revised this view, demonstrating that certain equol-producing bacteria harbor all four enzymes necessary to catalyze the complete conversion of daidzein to S-equol within a single organism (Gong et al. 2023). This finding suggests that equol production can be an intrinsic metabolic capability of specific bacterial species rather than a community-level cooperative process.

Advances in shotgun metagenomic sequencing have further expanded our understanding of equol-producing microbiota by enabling species-level resolution of functional pathways. Using DNA shotgun metagenomics, at least nine bacterial species with the genetic potential to produce equol have been identified, highlighting a greater diversity of equol-producing taxa than previously recognized through culture-dependent approaches alone (Dufault-Thompson et al. 2022). These findings provide an important genomic framework for interpreting culture-based isolation studies and for evaluating whether newly isolated strains possess the complete enzymatic machinery required for S-equol biosynthesis.

There is notable variability among individuals in the metabolism of daidzein, with roughly 30–60% of Asians and 25% of Western populations able to convert daidzein into S-equol (Setchell and Cole 2006). This variability is primarily linked to differences in the composition of the gut microbiota (Rowland et al. 2000; Possemiers et al. 2007). Humans display notable interindividual variability in S-equol production, a phenomenon not seen in other species. In contrast, the gut microbiome of rats and mice reliably converts daidzein to S-equol (Juniewicz et al. 1988; Ward et al. 2005). Studies have shown that bacteria are enantioselective in this process, exclusively forming S-(-)equol rather than R-(+)equol. Research also suggests that S-equol production is closely associated with specific gut microbiota and the intestinal environment conducive to equol formation (Vázquez et al. 2017; Iino et al. 2019). The specific relationship between the presence of S-equol-producing bacteria and an individual’s ability to synthesize S-equol is not yet fully elucidated, necessitating further research.

The aim of the present study was therefore, to isolate and identify aerobic and anaerobic microbes capable of metabolizing daidzein to S-equol using simple media like nutrient and De Man-Rogosa-Sharpe (MRS) agar from feces and intestine of albino Wistar rats. Achieving successful isolation of these microbes could reveal additional strains with potential implications for human health. This may open avenues for future therapeutic strategies and personalized nutritional interventions.

## Material and methods

### Chemicals and materials

The reference standard of daidzin was purchased from TCI chemicals, Tokyo, Japan. daidzein (CAS 486–66-8) and S-equol (CAS 531–95-3) were purchased from Tocris Biosciences, Bristol, UK. All the chemicals and solvents used were analytical grade. Toluene, ethyl acetate, formic acid, acetic acid, and methanol were procured from Merck, Mumbai, India. Pre-coated silica gel aluminum plates 60 F254 were purchased from Merck, Darmstadt, Germany. *Rhizopus oryzae* NCIM 1299 culture was procured from National Collection of Industrial Microorganisms, National Chemical Laboratories, Pune, India.

### Culture and maintenance of *R. oryzae*

Primary culturing of the fungus was performed on potato dextrose agar (PDA) by sprinkling the freeze-dried culture directly onto plates to obtain pure colonies (Fig. S2a). Mature colonies typically required 5–10 days on PDA for complete growth and sporulation. For maintenance, *R. oryzae* was sub-cultured on PDA slants and stored at 4 °C (Fig. S2b). For inoculum preparation, the fungus was transferred into potato dextrose broth (PDB) and incubated at 28 °C and 37 °C for 24 h (Fig. S2c). Under these liquid culture conditions, *R. oryzae* rapidly reached exponential growth phase, which was confirmed by microscopic examination of actively growing hyphae (Fig. S2d). The exponential-phase culture was then used as the seed inoculum for soybean fermentation.

### Fermentation of soybean seeds by *R. oryzae*

Soybean was soaked overnight, dehulled, and broken into halves, then packed and sealed in autoclavable plastic bags. The samples were sterilized at 121 °C/15 psi for 15 min and cooled completely before inoculation. Fermentation was initiated by aseptically adding 10% (w/v) *R. oryzae* seed culture prepared as described above. The inoculated bags were incubated at 28 °C for 48 h to allow fermentation.

### Preparation of soybean diet

**Fig. 2:**
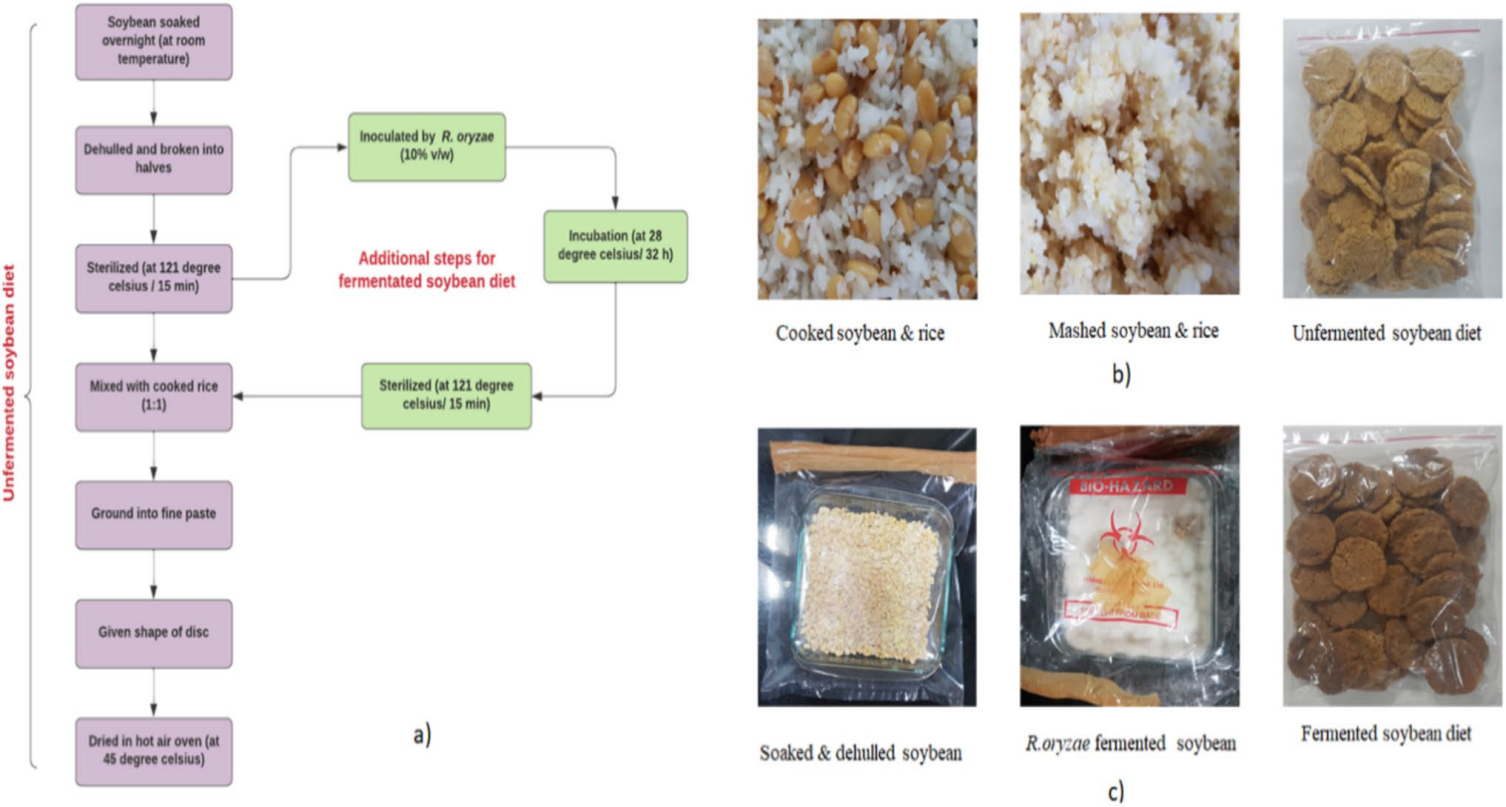
Soybean diet for enrichment of gut microbiota in albino Wistar rats. **a** Flow diagram illustrating the preparation process of two types of soybean diets, showcasing the diet formulation and specific ingredients used for each diet, as well as the sequential steps involved the preparation. **b** Unfermented soybean diet, **c** Fermented soybean diet

Two types of soybean feed were prepared: unfermented soybean feed and fermented soybean feed (Fig. 2). For the unfermented soybean feed, soybean and rice were mixed in 1:1 (w/w) ratio, cooked thoroughly, and then ground into a smooth paste. The paste was shaped into discs, forming biscuits and dried in a hot air oven at 50 °C until completely dried. The preparation of the fermented soybean feed began with soaking soybeans overnight at room temperature. The soybeans were then dehulled, split into halves. The soybeans were then sterilized at 121 °C for 15 min in autoclave-safe plastic bags followed by inoculation with 10% (v/w) of *R. oryzae*. The mixture was left to ferment at 28 °C for 48 h in an incubator. After fermentation, it was sterilized again at 121 °C for 15 min, mixed with cooked rice in a 1:1 (w/w) ratio, ground into a paste, shaped into discs, and dried in a hot air oven at 50 °C.

## Experiments on animals

### Ethical statement

This research project received official approval from the Institutional Animal Ethics Committee (IAEC) of Jamia Hamdard, New Delhi, under proposal number 1476. The study was meticulously planned to adhere to the ethical standards and protocols mandated by the IAEC. Additionally, all aspects of animal care, handling, and experimental procedures were conducted in strict compliance with the guidelines established by the Committee for the Purpose of Control and Supervision of Experiments on Animals (CPCSEA), New Delhi, India. These guidelines are designed to ensure the humane and ethical treatment of animals used in research, reflecting a strong commitment to responsible scientific practices.

### Animal handling

Albino Wistar rats weighing approximately 250 g (± 20 g) were utilized in the study. The rats were housed in air-conditioned facilities maintained at ± 24 °C within polypropylene cages. They were exposed to an alternating 12-h light/dark cycle and were provided with rat chow pellets and ad libitum access to drinking water. A 10-day acclimation period was allowed prior to the start of the experiments to minimize stress and ensure stable baseline conditions ( Debnath et al. 2024; National Research Council 2011).

### Enrichment of gut microbiota and sample collection

A dietary intervention study was conducted to enrich the gut microbiota of healthy Wistar rats with microbes capable of metabolizing soy isoflavones. The study involved two groups, each containing two rats. Over a period of 15 days, both groups were maintained on a strict soybean diet and provided with unlimited access to drinking water. Group I was given an unfermented soybean feed, which was rich in daidzin, while Group II received a fermented soybean feed, high in daidzein. On the 10th day, the rats were placed in two metabolic cages, with each cage housing the two rats from one group. The animals were maintained under the same environmental conditions (24 ± 1 °C) throughout the metabolic study. Fecal and urine samples were collected daily from days 11 to 15. At the end of the experiment, the rats were sacrificed, and microbes were isolated from their guts.

### Extraction of S-equol

To extract S-equol, the method was adapted from Xu et al. 1994, with slight modifications. Initially, 5 g of fermented soybean slurry was mixed with 20 mL of ethyl acetate. This mixture was then vigorously agitated using a vortex mixer at room temperature for 20 min to facilitate thorough mixing. After agitation, the sample was centrifuged at 2000 g for 5 min to separate the ethyl acetate layer, which was then carefully collected. The collected extract was evaporated to dryness, and the residue was re-dissolved in 2 mL of 80% methanol. The final solution was filtered through a 0.2-μm membrane filter to remove any particulate matter, making it suitable for further analysis.

### Analysis of S-equol

#### High performance thin layer chromatography for detection of S-equol

High-performance thin-layer chromatography (HPTLC) was conducted on pre-coated silica gel aluminum plates (60 F254, 10 × 10 cm, with a layer thickness of 0.2 mm) from E. Merk, KGaA, Darmstadt, Germany. Prior to use, the plates were washed with methanol, allowed to air dry, and then activated by heating at 110 °C for approximately 10 min. The mobile phase consisted of ethyl acetate, toluene, and formic acid in a volumetric ratio of 5:4:1. The chromatographic development was performed in a 10 × 10 cm twin trough glass chamber from Camag, Muttenz, Switzerland, which had been pre-saturated with the mobile phase. A deuterium lamp providing a continuous UV spectrum ranging from 190 to 400 nm was used for radiation. Densitometric scanning was carried out at a wavelength of 200 nm using a Camag TLC scanner III, operated in absorbance mode, and controlled by WinCATS software version 1.4.4 (Gangwar et al. 2024).

#### High performance liquid chromatography (HPLC) analysis

S-equol in dual fermented samples was estimated by HPLC analysis performed with Shimadzu HPLC system (model- SPD-10 AVP), Shimadzu corporation, Japan, equipped with quaternary pump and UV–VIS- detector A 20 µl volume injection was injected and separated over a C-18 column (Zorbax SB-C18, 4.6 × 50 mm, Agilent 5 µm particle size, made in Santa Clara, USA). The mobile phase consisted of 0.1% formic acid in water (solution A) and methanol and acetonitrile (2:1 v/v) (solution B). The column was maintained at a temperature of 25 °C, with a flow rate of 1 ml/min. The elution was isocratic, with a 50:50 ratio of solution A to solution B. S-equol was detected at 205 nm, with a run time of 20 min and a retention time of 6 min. The method was adapted with slight modifications from Li et al. (2018).

### Isolation of microbes

Microbes were isolated from rat feces and the intestine. The rats were killed under anesthesia with pentobarbital sodium salt (50 mg/kg body weight), and the intestines were collected. Intestines were immediately transferred into sterilized 100-mL Erlenmeyer flask containing 50 mL of normal saline and three acid-washed glass beads (3-mm diameter). After the mixtures had been vortexed well, serial dilution was carried out 5 times. Weighing 1 g of feces, it was mixed with 10 mL of normal saline and then serially diluted 5 times. Fecal and intestinal dilutions were plated on nutrient agar and MRS agar. The plates were then incubated at 37 ℃ for 24 h under aerobic and anaerobic conditions. Single colonies were transferred to respective agar slants under aerobic and anaerobic conditions. The anaerobic conditions were achieved in a desiccator, using calcium carbonate (CaCO_3_) at the bottom; sterile water was sprinkled on CaCO_3_ to initiate the release of carbon dioxide (CO_2_). Prior to sealing, a lit candle was introduced to consume any residual oxygen (O_2_) present in the desiccator. The sealing of the desiccator was accomplished using parafilm to effectively prevent the exchange of gases between the interior and exterior environments (Khan et al. 2020).

### Selection of microbe based on metabolite

Soybean was fermented consecutively by two microbes, first by *R. oryzae* (bioconversion of daidzin into daidzein) *and* then by isolated microbes (conversion of daidzin into S-equol).

#### Fermentation by isolated microbes

*R. oryzae* fermented soybean was ground into fine paste and soybean slurry was formed by adding distilled water to fermented soybean paste in the ratio 2:1. In an Erlenmeyer flask with a capacity of 100 ml, 50 g of slurry was placed and then sterilized at 121 °C/15 psi pressure for 15 min. The slurry was cooled and inoculated using loop full of each isolated microbe in different test tubes. Test flasks were incubated at 37 °C for 24 h under aerobic and anaerobic conditions as per the requirement of isolated microbe. S-equol was extracted according to Xu et.al, after complete fermentation. The extracted samples were analyzed by HPTLC and HPLC for the presence of equol; thus, identifying the equol-producing microbe.

### Molecular identification

#### PCR amplification of 16S rRNA

To prevent any laboratory contamination, the PCR was conducted using clones that were directly picked from the agar plate. The PCR mixture, with a total volume of 50 µL, consisted of 33 µL of nuclease-free water, a single colony picked from agar plate (1pick), 2 µL of the forward primer 16 s F (5′-AGAGTTTGATCCTGGCTCAG-3′) (10 µM), and 2 µL of the reverse primer 16 s R (5′-GGTTACCTTGTTACGACTT-3′) (10 µM), 10 µL of 10X reaction buffer, 2 µL of a dNTP mix (10 mM), and 1 µL of Taq DNA polymerase (2.5 U/µL). The amplification was carried out in a Master cycler® Thermocycler (DNAmp, Bhat Biotech). The amplification program consisted of one initial denaturation cycle at 94 °C for 10 min followed by 35 cycles of denaturation at 94 °C for 1 min, annealing at 56 °C for 1 min, extension at 72 °C for 1 min, and finally one cycle for final extension at 72 °C for 10 min.

### Gel extraction

The PCR products obtained from the 16S PCR reactions were purified to eliminate any unincorporated dNTPs and primers prior to sequencing. This purification process was carried out using a PCR purification kit (GENEASY GEL ELUTION KIT, Bhat Biotech India Pvt Ltd) as illustrated in Fig. S2. This step ensured that only the amplified DNA of interest was retained for subsequent sequencing, thereby enhancing the accuracy and reliability of the results.

### 16S rRNA sequence analysis

The 16S rDNA region was amplified using PCR, and both DNA strands were sequenced using an automated 3037xl DNA analyzer from Applied Biosystems. The sequencing was performed with the BigDye® Terminator v3.1 cycle sequencing kit, also from Applied Biosystems. The resulting sequence data were aligned and dendrograms were created using Sequence Analysis software version 5.2. Prior to conducting phylogenetic analysis, the sequences from the forward and reverse strands were aligned using suitable software. The 16S rRNA gene sequences obtained from isolates MG1, MG2, MG3, and MG4 were submitted to the NCBI GenBank database. The corresponding accession numbers are PX459562 (MG1), PX459563 (MG2), PX459564 (MG3), and PX459565 (MG4).

### Phylogenetic analysis

The sequences were compared to the non-redundant NCBI database using BLASTN under default settings to identify the most closely related sequences. The expected value and e-values for the closest matches were recorded. To further analyze the sequences, the ten most similar sequences were aligned using CLUSTAL W2. The resulting multiple alignment file was subsequently used to construct a phylogram using MEGA5 software.

## Results

### Isolation of microbes

A total of 48 culturable microbes were isolated from both groups of albino Wistar rats, of which 30 were from feces and 18 from intestine. All microbes isolated from the intestine were anaerobic; however, isolates from feces contained 13 aerobic and 17 anaerobic microbes. All four S-equol-producing microbes were anaerobic. MG1 (PX459562) and MG4 (PX459565) were isolated from the intestine of rats in Group II, MG2 (PX459563) was isolated from the intestine of a rat in Group I, and MG3 (PX459564) from feces of a rat in Group I. The morphology of strain MG1 showed pale yellow colonies accompanied by biofilm formation on nutrient agar, and Gram staining revealed gram-negative rod-shaped bacilli. The morphology of strain MG2 showed white to yellowish moist, smooth, and opaque colonies on nutrient agar, with pink gram-negative bacilli on Gram staining. The morphology of strains MG3 and MG4 showed spherical, smooth, opaque, creamy colonies on nutrient agar, and Gram staining revealed gram-positive round cocci in pairs and chains (Fig. S3).

Preliminary tests were performed to determine which rats excreted S-equol in their urine and feces. The rats from both groups excreted S-equol, with fecal levels consistently higher than urinary levels, irrespective of the diet. These findings underscore a significant correlation between diet and S-equol levels, as reported in prior research (Gangwar et al. 2024). The preliminary studies were essential to confirm that the experimental animals were metabolizing daidzein to S-equol, indicating that their gut harbored S-equol-producing microbes. This confirmation was necessary to proceed with isolating S-equol-producing microbes from the experimental animals.

Rats that were fed a fermented soy diet consistently exhibited higher levels of S-equol compared to those consuming an unfermented soy diet. This observed difference in S-equol levels can be attributed to the distinct substrates present in each type of diet. Specifically, the unfermented soy diet contains daidzin, while the fermented soy diet contains daidzein. Both daidzin and daidzein are crucial for the metabolic conversion process leading to the production of S-equol, which is carried out by specific gut bacteria. Daidzin, the predominant form in the unfermented diet, must first be converted to daidzein before it can be metabolized to S-equol. In contrast, the fermented diet provides daidzein directly, bypassing the initial conversion step. This direct availability of daidzein in the fermented diet enhances the efficiency of the metabolic process, leading to increased production of S-equol. Despite the presence of S-equol in the biological samples from both groups of rats, variations in S-equol concentrations suggest that the availability of daidzein a critical precursor for S-equol production fluctuates. These findings offer valuable insights into the complex interactions between dietary components and gut microbiota. They underscore the significant role of dietary choices in shaping microbial metabolism, which in turn has implications for overall health outcomes. The study highlights how dietary modifications can influence gut microbiota activity and, consequently, affect the production of bioactive compounds like S-equol, with potential implications for health and disease management.

### Fermentation of soybean by *R. oryzae*

The total isoflavone content of soybean, measured using the aluminum chloride colorimetric method (Chang et al. 2002) with quercetin as a standard, was 47.89 ± 2.86 µg/g, of which daidzin accounted for 16.55 ± 3.01 µg/g. After fermentation for 48 h, the daidzin content was reduced to 8.46 ± 3.62 µg/g as it was biotransformed into daidzein by the action of β-glucosidase enzyme produced by *R. oryzae*, which acts upon the sugar moiety of glycosidic soy isoflavones, converting them into aglycone molecules. The percentage conversion of daidzin to daidzein was found to be 51%.

### S-equol analysis

**Fig. 3:**
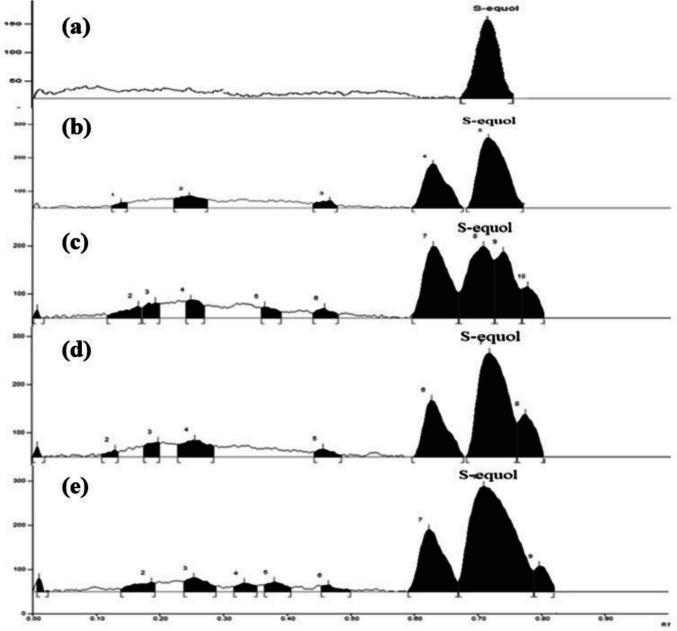
HPTLC chromatogram showing S-equol peak **a** S-equol standard (100 ng/ml); **b** MG1—*C. freundii*; **c** MG2—*E. fergusonii*; **d** MG3—*E. faecalis*; and **e** MG4 *E. faecalis*

A total of 48 samples were analyzed by HPTLC. Quantification of S-equol was achieved using calibration curves constructed from pure standards sourced from Tocris Biosciences, Bristol, UK. A 100 μg/mL stock solution was carefully prepared by dissolving 1 mg of S-equol standard in 10 mL of methanol. The calibration curve was established by applying varying amounts (0.5, 0.6, 0.7, 0.8, 0.9, and 1.0 μL) of this stock solution to HPTLC plates in triplicate, yielding concentrations from 50 to 100 ng/band. Equol concentration in the cultures was determined using a linear standard curve (Fig. S4), which adhered to the equation *y* = 25.386*x*−32.429, where *y* represents the HPTLC peak area and *x* denotes the equol concentration. S-equol peaks were observed in four samples: MG1, MG2, MG3, and MG4 (as illustrated in Fig. 3). The corresponding S-equol concentrations were 7.53 ± 0.74, 5.90 ± 1.20, 6.85 ± 1.12, and 6.90 ± 1.84 g/g of fermented soybean, respectively. The separation of S-equol from the sample matrix occurred at an R_f_ value of 0.77.

**Fig. 4:**
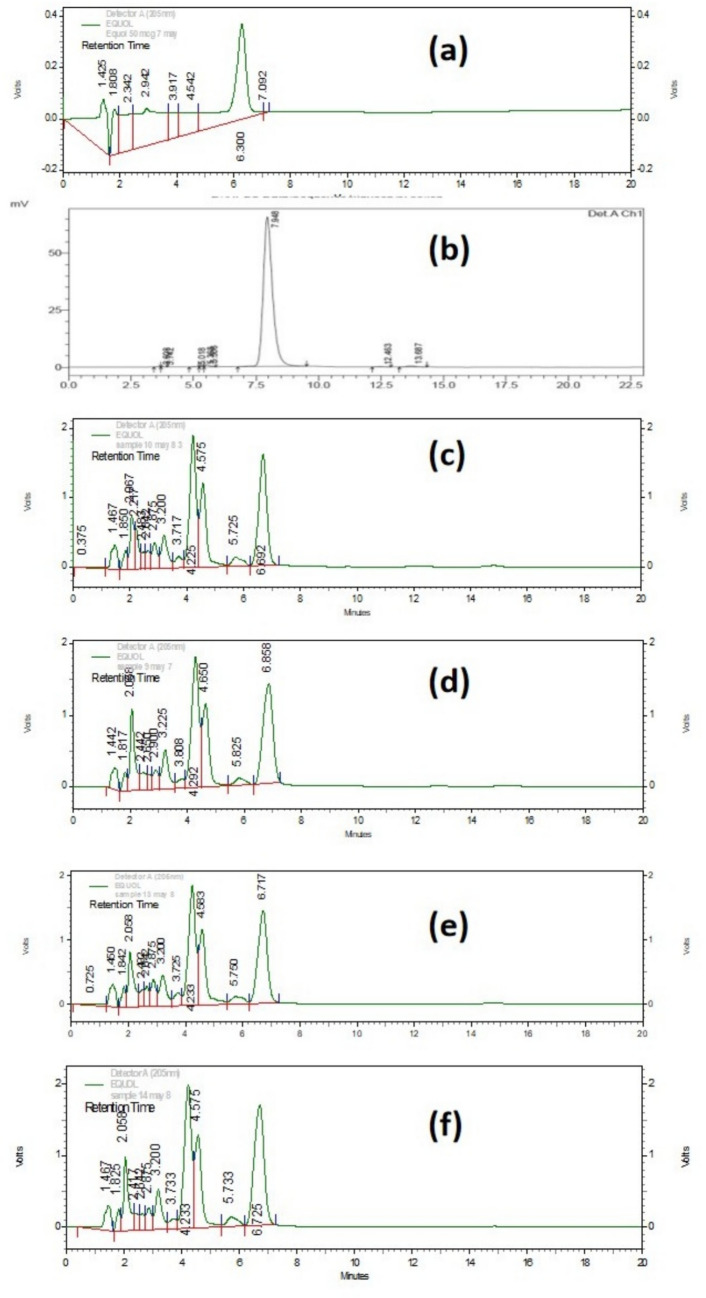
HPLC chromatogram showing elution of S-equol **a** S-equol standard (50 µg/ml); **b** Daidzein standard; **c** MG1-* C. freundii*; **d** MG2—*E. fergusonii*; **e** MG3—*E. faecalis*; and **f** MG4 *E. faecalis*

Four samples previously analyzed by HPTLC were subjected to further quantification via HPLC as shown in Fig. 4. The HPLC analysis revealed that S-equol eluted at retention time of 6.3 min in the chromatographic system. Calibration curves for S-equol quantification were established using pure standards sourced from Tocris Biosciences, Bristol, UK, covering concentrations ranging from 10 to 100 μg/mL. The concentration of equol in the cultures was calculated according to a linear standard curve Fig. S5, which followed the equation *y* = 205729*x* + 2E + 06 between the HPLC peak area (*y*) and the concentration of equol (*x*). The concentrations of S-equol produced by pure cultures of strain MG1, MG2, MG3 and MG4 were 7.56 ± 0.49, 5.95 ± 0.84, 6.83 ± 0.74, and 6.91 ± 0.48 μg/g of the fermented soybean.

### Microbe identification

**Fig. 5:**
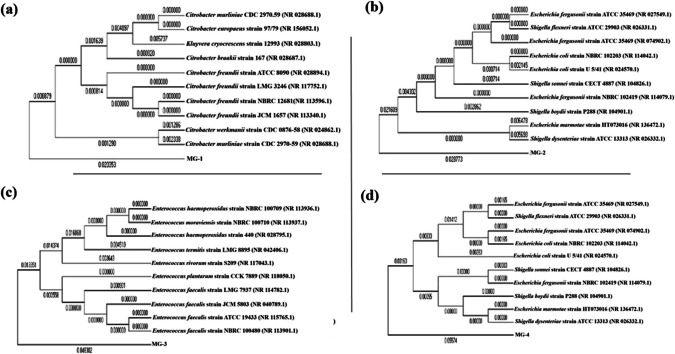
Phylogenetic tree analysis of 16S rRNA gene sequences of microbial strains **a** MG1—*C. freundii*; **b** MG2—*E. fergusonii*; **c** MG3—*E. faecalis* and **d** MG4—*E. faecalis*

The four bacterial strains, MG1, MG2, MG3, and MG4, underwent detailed characterization via near-complete 16S rRNA gene sequencing, as shown in Fig. S6. The 16S rRNA gene sequences were submitted to the NCBI GenBank database under accession numbers PX459562 (MG1), PX459563 (MG2), PX459564 (MG3), and PX459565 (MG4). Sequence analysis revealed a remarkable degree of similarity to known cultured bacterial species: 99% similarity for both MG1 and MG3, 98% for MG2, and 97% for MG4. Phylogenetic analysis further clarified the relationships among these strains, indicating that strain MG1 is closely related to *C. freundii* strain ATCC 8090. Meanwhile, MG2 is associated with *E. fergusonii* strain NBRC 102419. Additionally, both MG3 and MG4 align closely with *E. faecalis* strain NBRC 100480. These phylogenetic relationships are visually represented in Fig. 5 and elaborated in Supplementary Tables 1–4.

## Discussion

Dietary modifications are pivotal in reshaping gut microbiota composition and influencing the metabolism of isoflavones, especially in individuals harboring equol-producing bacteria. Research underscores that a variety of factors—including dietary patterns, the food matrix, gut microbiota profiles, bacterial fermentation processes, intestinal transit time, and the redox state of the large intestine—play significant roles in determining the metabolism, bioavailability of isoflavones, and equol production (Decroos et al. 2005). Recent advances have further highlighted the critical role of gut microbiota in modulating isoflavone bioavailability, where specific bacteria such as *Bifidobacterium* spp., *Eubacterium* spp., and *Adlercreutzia* spp. metabolize soybean isoflavones into equol and other active metabolites, enhancing their biological effects (Huang et al. 2022). Notably, diets rich in carbohydrates have been found to enhance equol production in those with the appropriate gut microbiota, illustrating the complex interplay between diet and gut microbiota in shaping metabolic outcomes. This intricate relationship between diet and gut microbiota suggests that personalized dietary interventions could be leveraged to boost the production of beneficial compounds like equol. By tailoring dietary choices to align with an individual’s unique microbial composition, it may be possible to enhance the bioavailability and efficacy of isoflavones, thereby promoting better health outcomes. This understanding opens the door to more precise dietary strategies that support the natural synthesis of health-promoting compounds in those with suitable gut microbiota. Consequently, dietary strategies represent a promising avenue for optimizing isoflavone metabolism and equol production, particularly for individuals predisposed to equol production (Decroos et al. 2005; Huang et al. 2022).)

The fermentation of carbohydrates by the gut microflora is a crucial process that leads to the generation of various byproducts, including hydrogen gas and short-chain fatty acids. These compounds play important roles in various physiological processes within the host. A seminal study conducted by Setchell and colleagues in 2005 shed light on the specific role of hydrogen gas in the production of equol, a metabolite derived from the soy isoflavone daidzein (Setchell et al. 2005). The researchers proposed that hydrogen gas acts as a vital electron donor, facilitating the biotransformation of daidzein into equol. This discovery underscored the importance of hydrogen gas in the equol production mechanism. Furthermore, the study also revealed that increased equol production was associated with the presence of propionate and butyrate, two SCFAs produced during carbohydrate fermentation. This observation suggests that diets rich in carbohydrates may have the potential to promote equol synthesis, likely by providing the necessary substrates and creating a favorable gut environment for equol-producing microbes. Building upon these findings, the researchers in the current study aimed to enrich the gut microbiome of albino Wistar rats by administering a soy-based diet for 15 days. To further support S-equol production, the diet was supplemented with rice as an additional carbohydrate source. After the dietary intervention period, the researchers observed a significant increase in S-equol excretion, indicating successful colonization of soy isoflavone-metabolizing bacteria within the gut. Interestingly, while the rats fed fermented diets exhibited higher levels of S-equol excretion, the differences were not statistically significant. Nevertheless, by the end of the study, both groups of rats showed an overall increase in S-equol excretion, demonstrating the efficacy of the dietary approach in promoting equol production (Setchell et al. 2005; Liang et al. 2024).

The metabolism of bioactive dietary compounds by the gut microbiome can significantly modify intestinal activities, thereby influencing host health. In vitro research has demonstrated that S-equol, a metabolite of daidzein, exhibits higher biological activity compared to its precursor, leading to extensive investigation into its clinical properties. Equol, with its heterocyclic structure and two phenoxyl groups, is known for its ability to scavenge superoxide anions and hydroxyl radicals. It also possesses a central furan ring with relatively inert oxygen. Uniquely, equol has a chiral center at the C3 position, existing as two enantiomers: R-equol and S-equol (Wang et al. 2005). Both enantiomers are bioavailable and exhibit estrogenic activity, but R-equol, which binds to the estrogen receptor ER-α, is considered a weak estrogen, whereas S-equol, with a high affinity for ER-β, displays stronger estrogenic activity. Recent studies emphasize the importance of distinguishing these enantiomers through advanced analytical techniques for therapeutic applications (Okami et al. 2025).

In the present study, the analysis of fermented soybean using HPTLC and HPLC confirmed the production of S-equol. The quantification of S-equol by HPLC was successful, providing reliable measurements of its concentration in the cultures. However, the presence of R-equol alongside S-equol was not determined and remains an area for further investigation, which could be addressed through chiral phase HPLC or LCMS.

Given that S-equol is solely produced by intestinal bacteria, the isolation and identification of daidzein-metabolizing and equol-producing microorganisms are essential steps toward enhancing S-equol production. This advancement could significantly expand the therapeutic potential of S-equol in human health. Future research should prioritize the precise characterization of both S-equol and R-equol production, while also assessing any potentially harmful or toxic byproducts generated by these microbial strains. To achieve this, the application of advanced analytical techniques—such as chiral phase HPLC, LC–MS, NMR spectroscopy, and high-resolution mass spectrometry (HR-MS)—is crucial (Okami et al. 2025). These methods will enable accurate profiling of these metabolites and offer comprehensive insights into their safety and efficacy, ensuring that any therapeutic applications are both effective and safe.

Several bacteria capable of producing equol have been discovered. One such strain is SNU-Julong 732, which was isolated from human feces. This particular strain has been proven to efficiently convert dihydrodaidzein into equol, indicating its ability to contribute to equol production through microbial processes (Minamida et al. 2006). Additionally, a mixed culture derived from human feces, rather than individual bacterial strains, has been found to transform daidzein into equol (Decroos et al. 2005). In 2006, Minamida et al. (Minamida et al. 2006) reported a bacterial strain isolated from rat feces as the only one capable of directly converting daidzein into equol. Most of the identified equol producing bacteria have been obtained from samples of human and rat intestines. Moreover, a recent 2025 study in school-age children identified specific bacterial species, such as *Asaccharobacter celatus* and *Slackia isoflavoniconvertens*, as more abundant in equol producers than non-producers. These findings reveal inter-individual microbial diversity as a determinant of equol-producing capacity, emphasizing the potential for personalized dietary interventions to optimize isoflavone metabolism and health benefits (Wada et al. 2025).

Recent advances in equol research have led to a reassessment of the microbial mechanisms underlying daidzein biotransformation. While earlier studies suggested that equol production may result from the coordinated activity of multiple bacterial species within the gut ecosystem, more recent reviews indicate that certain equol-producing bacteria harbor all four enzymes required for the complete conversion of daidzein to S-equol within a single organism (Gong et al. 2023). This emerging paradigm aligns with findings from shotgun metagenomic studies, which have identified at least nine bacterial species possessing the genetic potential for equol biosynthesis, thereby expanding the known diversity of equol-producing taxa beyond those identified through culture-dependent methods alone (Dufault-Thompson et al. 2022). Together, these studies highlight that equol production can be an intrinsic metabolic capability of specific bacterial species rather than solely a community-driven process.

In the present study, our approach was to isolate single bacterial strains capable of producing S-equol**,** in line with recent evidence suggesting that equol biosynthesis can occur within individual microorganisms possessing the complete enzymatic pathway. Using simple culture media under both aerobic and anaerobic conditions, we successfully isolated four S-equol–producing anaerobic strains from the rat intestine. The phenotypic analysis placed strains MG1 (PX459562) and MG2 (PX459563) in the family *Enterobacteriaceae* and strains MG3 (PX459564) and MG4 (PX459565) in the family *Enterococcaceae.* The identity of the isolated strains was determined by comparing the sequence of the 16S rRNA gene with a database. The first isolate MG1 (PX459562) had 99% sequence similarity with *C. freundii* strain ATCC 8090. The second isolate MG2 (PX459563) had 98% sequence similarity with *E. fergusonii* strain NBRC 102419. The third and fourth isolates MG3 (PX459564) and MG4 (PX459565) had 99% and 97% similarity with *E. faecalis* strain NBRC 100480, respectively. Most of equol-producing microbes are classified within the family *Coriobacteriaceae*. To our knowledge, this is the first report of S-equol–producing bacteria belonging to the family *Enterobacteriaceae*. Additionally, it has been previously reported that another microbe, *E. faecium*, from the family *Enterococcaceae*, can produce equol in a mixed culture (Patel et al. 1998). *E. faecalis* and *E. faecium* share 97.3% homology values in 16 s rRNA sequences. Strain MG1 exhibited the highest S-equol production and was found to directly convert daidzein to S-equol. However, further investigation is required to fully understand the biotransformation mechanism of the remaining three strains.

Notably, the isolated strains demonstrated S-equol production without the addition of media supplements known to enhance production. This simple, cost-effective, and instrumentally undemanding process suggests potential for easy commercialization. However, strain MG2 (PX459563), which matches pathogenic *E. fergusonii*, should not be used for in vitro production of S-equol for therapeutic purposes. On the other hand, strains MG1 (PX459562), MG3 (PX459564), and MG4 (PX459565) match common commensal bacteria (*C. freundii* and *E. faecalis*) present in healthy individuals. While these strains hold promise for in vitro S-equol production, further research is essential to establish their safety and efficacy for such purposes.

## Conclusion

In conclusion, the successful isolation and characterization of S-equol-producing bacteria from rat intestines represent a significant advancement in gut microbiota research and its application in functional food development and therapeutic interventions. Importantly, the isolation of single bacterial strains capable of producing S-equol supports recent evidence suggesting that equol biosynthesis can occur within individual microorganisms rather than requiring coordinated activity among multiple taxa. Future research should prioritize mechanistic studies to fully elucidate the biotransformation mechanisms employed by the isolated strains MG1 (PX459562), MG3 (PX459564), and MG4 (PX459565), as understanding these pathways will optimize conditions for maximal S-equol production. Comprehensive safety and efficacy assessments of the isolated strains are imperative before considering them for therapeutic purposes, including evaluating their potential pathogenicity, particularly for strains closely related to known pathogens like MG2 (PX459563, *E. fergusonii*). The demonstrated simple and cost-effective process of S-equol production by these strains holds promise for commercial applications, with scaling up production and ensuring consistency and purity being crucial steps towards commercialization. Investigating the clinical benefits of S-equol, given its higher biological activity and specific estrogenic properties, could open new avenues for its use in health and medicine, such as managing menopausal symptoms, osteoporosis, and other estrogen-related conditions. Furthermore, exploring the potential for microbiome engineering to enhance S-equol production in situ within the human gut could offer a novel approach to improving health outcomes related to isoflavone metabolism. Detailed genetic and metabolic profiling of the isolated strains will provide a deeper understanding of their functional capabilities and interaction with host physiology, aiding in identifying key genes and enzymes involved in S-equol biosynthesis, thus fully harnessing the potential of these findings.

## Future work

A key limitation of the present study is that, although the isolated microbes MG1 (PX459562), MG2 (PX459563), MG3 (PX459564), and MG4 (PX459565) successfully produce S-equol, the identity of other by-products formed alongside S-equol remains unknown. It is crucial to ensure that these strains do not generate any toxic or harmful metabolites that could impact their safety and efficacy. Future studies should therefore focus on comprehensive metabolomic profiling of these strains. Employing advanced analytical techniques, such as chiral-phase HPLC, LC–MS, NMR spectroscopy, and high-resolution mass spectrometry (HR-MS), will enable detailed characterization of the metabolic profiles, including confirmation of the stereochemistry of equol (S- versus R-enantiomer) and identification of any additional metabolites.

Additionally, this study was conducted with a small sample size (*n* = 4) and in rats, which may limit reproducibility and human applicability. Future work should include larger cohorts and investigate whether these bacterial strains can colonize the human gut and produce S-equol under similar conditions. The enzymatic pathways used by MG3 and MG4 to convert daidzein into S-equol remain uncharacterized; genomic, transcriptomic, and proteomic analyses will be valuable in elucidating these mechanisms, including determining whether these strains harbor the complete set of enzymes required for S-equol biosynthesis. Furthermore, assessing reproducibility of S-equol production across replicates and time points will be critical to ensure consistency and reliability.

Together, these future investigations will provide a comprehensive understanding of the metabolic capabilities, safety, and mechanistic pathways of these S-equol-producing bacteria, supporting their potential application in functional foods, therapeutics, and translational research.

## Supplementary Information

Below is the link to the electronic supplementary material.

ESM 1 (DOCX 2.29 MB)

## Data Availability

All nucleotide sequence data generated in this study have been deposited in the NCBI GenBank database under the accession numbers PX459562–PX459565 corresponding to isolates MG1–MG4. The sequences are publicly accessible through the GenBank repository. The S-equol–producing strains MG1, MG3, and MG4 are available to the research community upon reasonable request from Dr. Bibhu Prasad Panda (Microbial and Pharmaceutical Biotechnology Laboratory, Department of Pharmacognosy & Phytochemistry, School of Pharmaceutical Education & Research, Jamia Hamdard, New Delhi–110062, India) to support reproducibility and independent verification of the results.
