# Supplementary material for: S-equol producing bacteria: isolation and identification from Albino Wistar rat gut microbiota
Source: Appl Microbiol Biotechnol. 2026 Mar 2;110:86. doi: 10.1007/s00253-026-13759-4 (PMC12953384; doi:10.1007/s00253-026-13759-4)
Supplement: Supplementary file 1 [file 253_2026_13759_MOESM1_ESM.docx]

**S-Equol Producing Bacteria: Isolation and Identification from Albino Wistar Rat Gut**

**Microbiota**

**Megha Gangwar^1,2^, Sanaa Ismael Abduljabbar^2^, Mohammad Sarwar Alam^3^, Kahksha Ahmed^4,^ Sameena Naaz^5^*, Bibhu Prasad Panda^2^***

^1^Department of Food Technology, School of Interdisciplinary Sciences & Technology, Jamia Hamdard, New Delhi-110062, India

^2^Microbial and Pharmaceutical Biotechnology Laboratory, Department of Pharmacognosy & Phytochemistry, School of Pharmaceutical Education & Research, Jamia Hamdard, New Delhi-110062, India

^3^Department of Chemistry, School of Chemical and Life Sciences, Jamia Hamdard, New Delhi-110062, India

^4^Department of Computer Science and Engineering, St. Andrews Institute of Technology & Management (SAITM), Gurugram 122506, India.

^5^Department of Computer Science, School of Arts, Humanities and Social Sciences, University of Roehampton, London SW15 5PH, UK.

Megha Gangwar and Sanaa Ismael Abduljabbar have equal contribution.

**^*^Correspondence:**

Prof. Bibhu Prasad Panda, (M. Pharm., PhD), Microbial and Pharmaceutical Biotechnology Laboratory, Department of Pharmacognosy & Phytochemistry, School of Pharmaceutical Education & Research, Jamia Hamdard, New Delhi-110062, India. Email:

bppanda@jamiahamdard.ac.in; Tel.: +91 9990335013

**Supplementary Figures and Tables**

**Supplementary Figures**

**Figure S1**. Culture of *R. oryzae* : a) Potato dextrose agar plates b) Potato dextrose agar slants c) Potato dextrose broth d) Under microscope 40 (X)

**Figure S2:** a) DNA Gel electrophoresis of amplicons of MG1- *Citrobacter freundii*; B)MG2 - *Escherichia fergusonii* ; MG3 - *Enterococcus faecalis* and MG4 *Enterococcus faecalis .*Gel elution of 16S rRNA sequence analysis showing amplicon purification. This gel elution image displays the results of 16S rRNA sequence analysis, specifically the purification of amplicons. The gel electrophoresis technique was employed to separate and purify the amplified 16S rRNA fragments from the background noise.

**Figure S3.** Pure microbial culture and staining of s-equol producing microbes isolated from albino wistar rats, (a) MG1- Gram-negative bacilli-; (b) MG2- Gram-negative bacilli; (c) MG3- Gram-positive cocci-, and (d) MG4- Gram-positive cocci.

**Figure S4.** Calibration curve of S-equol (concentrations ranging from 50-100 ng) for HPTLC.

|  |  |
| --- | --- |
| 10 | 3692468 |
| 20 | 5540926 |
| 30 | 7811545 |
| 40 | 10467168 |
| 50 | 12822099 |
| 100 | 22019983 |

**Figure S5.** Calibration curve of S-equol (concentrations 10, 20, 30, 40, 50 and 100 μg) for HPLC.

**Figure S6.** Amplicons sequence of a) MG1- *Citrobacter freundii*; b) MG2 - *Escherichia fergusonii*; c) MG3 - *Enterococcus faecalis* and d) MG4 *Enterococcus faecalis.*

**List of Supplementary Table**

Table S1. GenBank accessions of the sequence analysis for MG1.

| **DESCRIPTION** | **Max**  **Score** | **Total**  **Score** | **E**  **Value** | **Score** | **Accession**  **Number** |
| --- | --- | --- | --- | --- | --- |
| *Citrobacter europaeus strain 97/79* | 1598 | 2037 | 0 | 99 % | NR_156052.1 |
| *Citrobacter werkmanii strain CDC 0876-58* | 1598 | 2251 | 0 | 99 % | NR_024862.1 |
| *Citrobacter freundii strain ATCC 8090* | 1592 | 2269 | 0 | 99 % | NR_028894.1 |
| *Citrobacter freundii strain LMG 3246* | 1587 | 2240 | 0 | 99 % | NR_117752.1 |
| *Citrobacter freundii strain NBRC 12681* | 1587 | 2264 | 0 | 99 % | NR_113596.1 |
| *Citrobacter murliniae strain CDC 2970-59* | 1587 | 2258 | 0 | 99 % | NR_028688.1 |
| *Citrobacter braakii strain 167* | 1587 | 2258 | 0 | 99 % | NR_028687.1 |
| *Citrobacter freundii strain JCM 1657* | 1585 | 2262 | 0 | 99 % | NR_113340.1 |
| *Kluyvera cryocrescens strain NBRC 102467* | 1576 | 2172 | 0 | 99 % | NR_114108.1 |
| *Kluyvera cryocrescens strain 12993* | 1576 | 2179 | 0 | 99 % | NR_028803.1 |

The sequence was compared with existing sequences in the NCBI database using the BlastN program. The score and expect values is presented in the table below. The DNA sequence of MG1 matches best with that of *Citrobacter freundii* strain ATCC 8090. A high score and low expect value indicates greatest homology.

Phylogenetic Tree MG1

| **ID** | **Description** | **% similarity** |
| --- | --- | --- |
| NR_028894.1 | *Citrobacter freundii strain ATCC 8090* | 99% |
| NR_117752.1 | *Citrobacter freundii strain LMG 3246* | 99% |
| NR_113596.1 | *Citrobacter freundii strain NBRC 12681* | 99% |

Table S2. GenBank accessions of the sequence analysis for MG2

| **DESCRIPTION** | **Max**  **Score** | **Total**  **Score** | **E**  **Value** | **Score** | **Accession**  **Number** |
| --- | --- | --- | --- | --- | --- |
| *Escherichia marmotae strain* HT073016 | 1788 | 2454 | 0 | 98% | NR_136472.1 |
| *Escherichia fergusonii strain* ATCC 35469 | 1788 | 2476 | 0 | 98% | NR_074902.1 |
| *Escherichia fergusonii strain* NBRC 102419 | 1788 | 2469 | 0 | 98% | NR_114079.1 |
| *Escherichia fergusonii strain* ATCC 35469 | 1788 | 2469 | 0 | 98% | NR_027549.1 |
| *Shigella flexneri strain* ATCC 29903 | 1788 | 2471 | 0 | 98% | NR_026331.1 |
| *Shigella sonnei strain* CECT 4887 | 1783 | 2465 | 0 | 98% | NR_104826.1 |
| *Escherichia coli strain* NBRC 102203 | 1781 | 2460 | 0 | 98% | NR_114042.1 |
| *Shigella boydii strain* P288 1777 | 2458 | 2458 | 0 | 98% | NR_104901.1 |
| *Escherichia coli strain* U 5/41 1773 | 2436 | 2436 | 0 | 98% | NR_024570.1 |
| *Shigella dysenteriae strain* ATCC 13313 | 1772 | 2456 | 0 | 98% | NR_026332.1 |

The sequence was compared with existing sequences in the NCBI database using the BlastN program. The score and expect values is presented in the table below. The DNA sequence of MG2 matches best with that of *Escherichia fergusonii* strain NBRC 102419. A high score and low expect value indicates greatest homology.

Phylogenetic Tree MG2

| **ID** | **Description** | **% Similarity** |
| --- | --- | --- |
| NR_136472.1 | *Escherichia marmotae strain* HT073016 | 98% |
| NR_074902.1 | *Escherichia fergusonii strain* ATCC 35469 | 98% |
| NR_114079.1 | *Escherichia fergusonii strain* NBRC 102419 | 98% |

Table S3. GenBank accessions of the sequence analysis for MG3

| **DESCRIPTION** | **Max Score** | **Total**  **Score** | **E**  **Value** | **Score** | **Accession**  **Number** |
| --- | --- | --- | --- | --- | --- |
| *Enterococcus faecalis strain* NBRC 100480 | 1160 | 1728 | 0 | 99% | NR_113901.1 |
| *Enterococcus faecalis strain* ATCC 19433 | 1160 | 1728 | 0 | 99% | NR_115765.1 |
| *Enterococcus faecalis strain* LMG 7937 | 1160 | 1723 | 0 | 99% | NR_114782.1 |
| *Enterococcus rivorum strain* S299 | 1138 | 1630 | 0 | 99% | NR_117043.1 |
| *Enterococcus moraviensis strain* NBRC 100710 | 1134 | 1581 | 0 | 99% | NR_113937.1 |
| *Enterococcus faecalis strain* JCM 5803 | 1134 | 1695 | 0 | 99% | NR_040789.1 |
| *Enterococcus haemoperoxidus* *strain* NBRC 100709 | 1133 | 1581 | 0 | 99% | NR_113936.1 |
| *Enterococcus plantarum strain* CCM 7889 | 1133 | 1577 | 0 | 99% | NR_118050.1 |
| *Enterococcus termitis strain* LMG 8895 | 1133 | 1579 | 0 | 99% | NR_042406.1 |
| *Enterococcus haemoperoxidus strain* 440 | 1129 | 1570 | 0 | 99% | NR_028795.1 |

The sequence was compared with existing sequences in the NCBI database using the BlastN program. The score and expect values is presented in the table below. The DNA sequence D5 (2) A/ sample 13/ MG3 matches best with that of *Enterococcus faecalis* strain NBRC 100480. A high score and low expect value indicates greatest homology.

Phylogenetic Tree MG3

| **ID** | **Description** | **% Similarity** |
| --- | --- | --- |
| NR_113901.1 | *Enterococcus faecalis strain* NBRC 100480 | 99% |
| NR_115765.1 | *Enterococcus faecalis strain* ATCC 19433 | 99% |
| NR_040789.1 | *Enterococcus faecalis strain* JCM 5803 | 99% |

Table S4. GenBank accessions of the sequence analysis for MG4.

| **DESCRIPTION** | **Max**  **Score** | **Total**  **Score** | **E**  **Value** | **Score** | **Accession**  **Number** |
| --- | --- | --- | --- | --- | --- |
| *Enterococcus faecalis strain* NBRC 100480 | 1323 | 1736 | 0 | 97% | NR_113901.1 |
| *Enterococcus faecalis strain* ATCC 19433 | 1323 | 1736 | 0 | 97% | NR_115765.1 |
| *Enterococcus faecalis strain* LMG 7937 | 1323 | 1730 | 0 | 97% | NR_114782.1 |
| *Enterococcus rivorum strain* S299 1301 | 1301 | 1301 | 0 | 96% | NR_117043.1 |
| *Enterococcus faecalis strain* JCM 5803 | 1297 | 1704 | 0 | 96% | NR_040789.1 |
| *Enterococcus moraviensis strain* NBRC 100710 | 1286 | 1286 | 0 | 96% | NR_113937.1 |
| *Enterococcus haemoperoxidus strain* NBRC 100709 | 1284 | 1284 | 0 | 96% | NR_113936.1 |
| *Enterococcus plantarum strain* CCM 7889 | 1284 | 1284 | 0 | 96% | NR_118050.1 |
| *Enterococcus termitis strain* LMG 8895 | 1284 | 1284 | 0 | 96% | NR_042406.1 |
| *Enterococcus silesiacus strain* R-23712 | 1273 | 1273 | 0 | 96% | NR_042405.1 |

The sequence was compared with existing sequences in the NCBI database using the BlastN program. The score and expect values is presented in the table below. The DNA sequence of MG4 matches best with that of *Enterococcus faecalis* strain NBRC 100480 . A high score and low expect value indicates greatest homology.

Phylogenetic Tree of MG4

| **ID** | **Description** | **% Similarity** |
| --- | --- | --- |
| NR_113901.1 | *Enterococcus faecalis strain* NBRC 100480 | 97 % |
| NR_115765.1 | *Enterococcus faecalis strain* ATCC 19433 | 97 % |
| NR_040789.1 | *Enterococcus faecalis strain* JCM 5803 | 96 % |
